# TGF-β1 stimulation and VDR-dependent activation modulate calcitriol action on skeletal muscle fibroblasts and Smad signalling-associated fibrogenesis

**DOI:** 10.1038/s41598-023-40978-w

**Published:** 2023-08-23

**Authors:** Ratchakrit Srikuea, Muthita Hirunsai

**Affiliations:** 1 Department of Physiology, Faculty of Science, Mahidol University, Bangkok, 10400 Thailand; 2 Department of Biopharmacy, Faculty of Pharmacy, Srinakharinwirot University, Ongkharak, Nakhon Nayok 26120 Thailand

**Keywords:** Cell biology, Physiology

## Abstract

Fibroblasts play a pivotal role in fibrogenesis after skeletal muscle injury. Excess fibrous formation can disrupt contractile functions and delay functional recovery. Although vitamin D receptor (VDR) is expressed explicitly in regenerating muscle compared with uninjured muscle, how calcitriol [1α,25(OH)_2_D_3_] directly regulates skeletal muscle primary fibroblast proliferation, the transition to myofibroblasts, and Smad signalling-associated fibrogenesis is currently unknown. Herein, the effects of calcitriol on cultured skeletal muscle primary fibroblasts of male C57BL/6 mice (aged 1 month old) were investigated. The percentage of BrdU^+^ nuclei in primary fibroblasts was significantly decreased after calcitriol treatment; however, the antiproliferative effect of calcitriol was diminished after TGF-β1 stimulation to induce fibroblast to myofibroblast transition. This suppressive effect was associated with significantly decreased VDR expression in TGF-β1-treated cells. In addition, *Vdr* siRNA transfection abolished the effects of calcitriol on the suppression of α-SMA expression and Smad2/3 signalling in myofibroblasts, supporting that its antifibrogenic effect requires VDR activation. Compared with calcitriol, the antifibrotic agent suramin could inhibit fibroblast/myofibroblast proliferation and suppress the expression of TCF-4, which regulates fibrogenic determination. Collectively, these findings suggest that profibrotic stimulation and VDR-dependent activation could modulate the effects of calcitriol on skeletal muscle fibroblast proliferation and fibrogenesis processes. Therefore, TGF-β1 and VDR expression levels are crucial determinants for the antifibrogenic effect of calcitriol on skeletal muscle after injury.

## Introduction

Skeletal muscle contributes critically to body functions. Due to its role in locomotor activity, skeletal muscle injury is one of the most common types of injury. After injury, cellular events involve degeneration, regeneration, and extracellular matrix (ECM) remodelling that coordinate the healing process of skeletal muscle. Muscle degeneration is an initial step in which inflammatory cells (e.g., neutrophils and macrophages) initiate inflammatory responses and the removal of tissue debris^1^. Following muscle degeneration, repair of damaged muscle fibres by skeletal muscle stem cells (SMSCs) occurs by incorporation into existing muscle fibres or fusion to form nascent muscle fibres^2^. For proper functional recovery, ECM remodelling that relies on the function of fibroblasts to produce connective tissue surrounding regenerating muscle fibres coincides with skeletal muscle regeneration to support the repair process and force transmission^3,4^. An imbalance between muscle regeneration and fibrous formation leads to fibrosis development, resulting in disrupted contractile functions and delayed functional recovery of skeletal muscle^5–7^.

Several lines of evidence suggest that skeletal muscle is a vitamin D-target tissue, while vitamin D and its receptor (vitamin D receptor, VDR) have diverse effects on skeletal muscle functions^8–12^, regenerative processes^13–15^, SMSC regulation^16^, myogenesis^17^, mitochondrial respiration^17,18^, and glycogen storage^19^. In addition, vitamin D supplementation has been demonstrated to improve the recovery of skeletal muscle after crush injury^20^ and exercise-induced muscle injury^21–23^. Nevertheless, an increase in prolyl-4-hydroxylase-β expression that plays an essential role in the synthesis of collagens after vitamin D supplementation^20^ and upregulation of ECM remodelling regulatory genes in hypertrophic muscle via overexpression of VDR have been reported^24^. These findings suggest the potential regulatory effect of vitamin D on skeletal muscle fibroblasts and ECM remodelling after muscle injury.

The regulation of ECM remodelling during the skeletal muscle repair process is primarily regulated by transforming growth factor-β (TGF-β)^5,25,26^. TGF-β signalling involves binding of the TGF-β ligand to the type I receptor (TβRI) and type II receptor (TβRII), which facilitates complex formation of TβRI and TβRII on the cell membrane. Then, TβRII phosphorylates TβRI at serine and threonine residues to induce activation of the Smad protein family to mediate the intracellular signal to the nucleus for regulation of ECM regulatory genes^27^. Dysregulation of the TGF-β/Smad signalling cascade through increased phosphorylation of Smad2/3 proteins induces accumulation of ECM components and subsequently forms fibrosis in dystrophic muscle that is susceptible to progressive degeneration and regeneration^6,28^. Therefore, proper activation of the TGF-β/Smad signalling cascade would improve the healing process and functional recovery after skeletal muscle injury.

Currently, the effect of calcitriol (the active form of vitamin D_3_) through its cognate receptor (VDR) on the TGF-β/Smad signalling cascade and fibrotic markers in fibroblasts has been reported in the lung^29^, liver^30^, and intestine^31^. However, no study to date has examined calcitriol action on skeletal muscle fibroblasts and the associated TGF-β/Smad signalling cascade, which plays a crucial role in fibrosis development after skeletal muscle injury. Therefore, the objective of this study was to investigate the effect of calcitriol on the regulation of skeletal muscle fibroblast proliferation, the transition to myofibroblasts, and Smad2/3 signalling-associated fibrogenesis. The antifibrogenic effect of calcitriol was compared with that of the antifibrotic agent suramin, which has been reported to inhibit fibrosis development and hasten functional recovery of skeletal muscle after injury^32^.

## Results

### Primary fibroblast characterization

**Figure 1:**
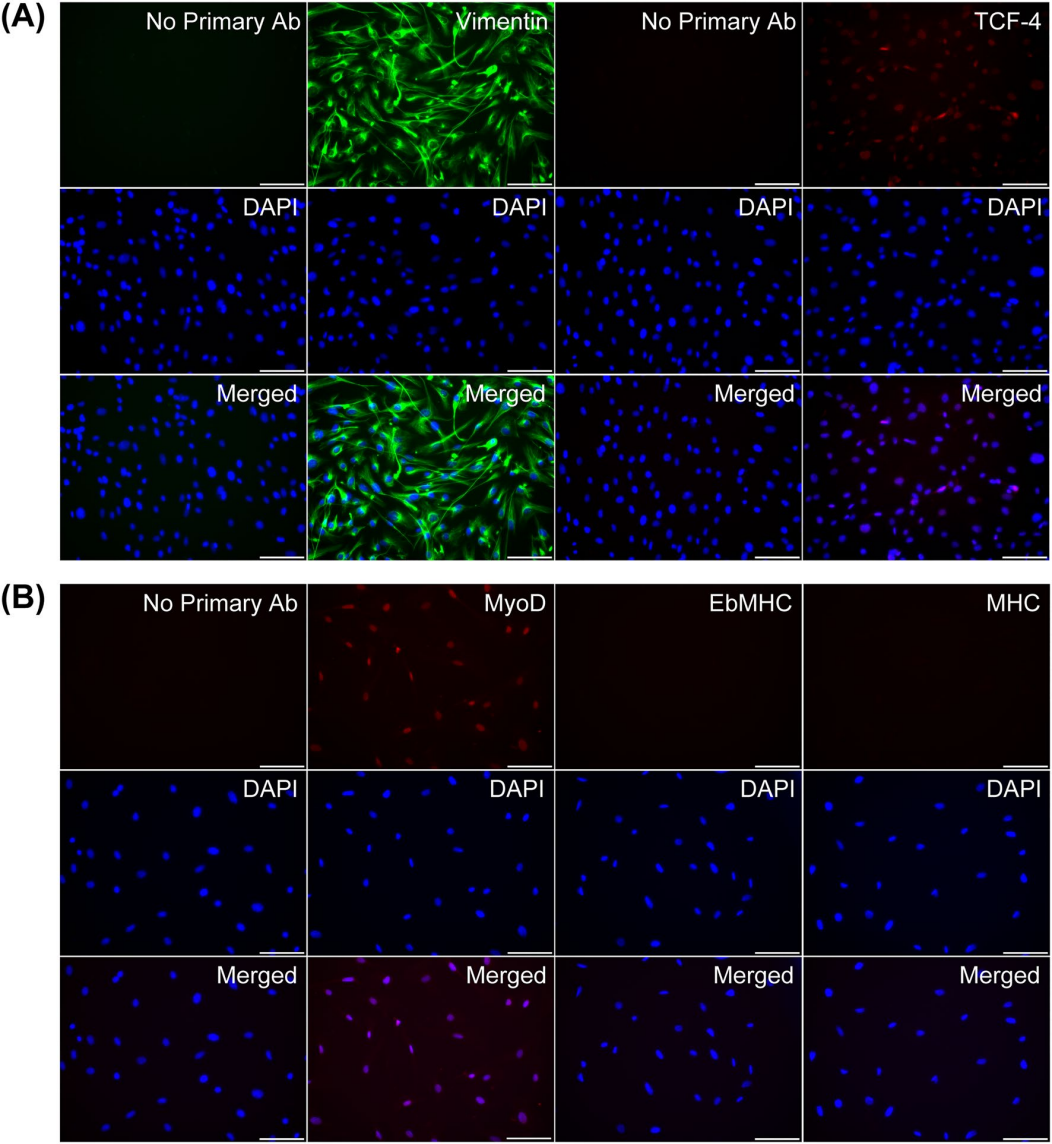
Primary fibroblast characterization. (**A**) Representative images of vimentin and TCF-4 protein expression in primary fibroblasts (passage 1) cultured in growth medium for 48 h. (**B**) Representative images of MyoD and myogenic differentiation marker (EbMHC and MHC) protein expression in primary fibroblasts after culture in myogenic differentiation medium for 48 h. Primary antibodies were omitted in the no primary antibody conditions. Images were taken at magnification × 200, scale bars = 100 µm.

Primary fibroblasts derived from skeletal muscle in this study were characterized based on vimentin^+^/TCF-4^+^ staining as illustrated in Fig. 1A. Cultured primary fibroblasts expressed the intermediate filament vimentin and demonstrated nuclear localization of the TCF-4 transcription factor, which regulates fibrogenic determination, as previously reported^33^. Due to the nature of primary fibroblasts and SMSCs that are derived from the mesodermal lineage and endogenously express MyoD, cultured primary fibroblasts were further verified as nondifferentiated skeletal muscle cells under culture in myogenic differentiation medium. Neither EbMHC nor MHC protein expression was detected after culture for 48 h, suggesting that this cell population was primary fibroblasts and not SMSCs (Fig. 1B).

### Calcitriol and suramin treatment decreased primary fibroblast proliferation

**Figure 2:**
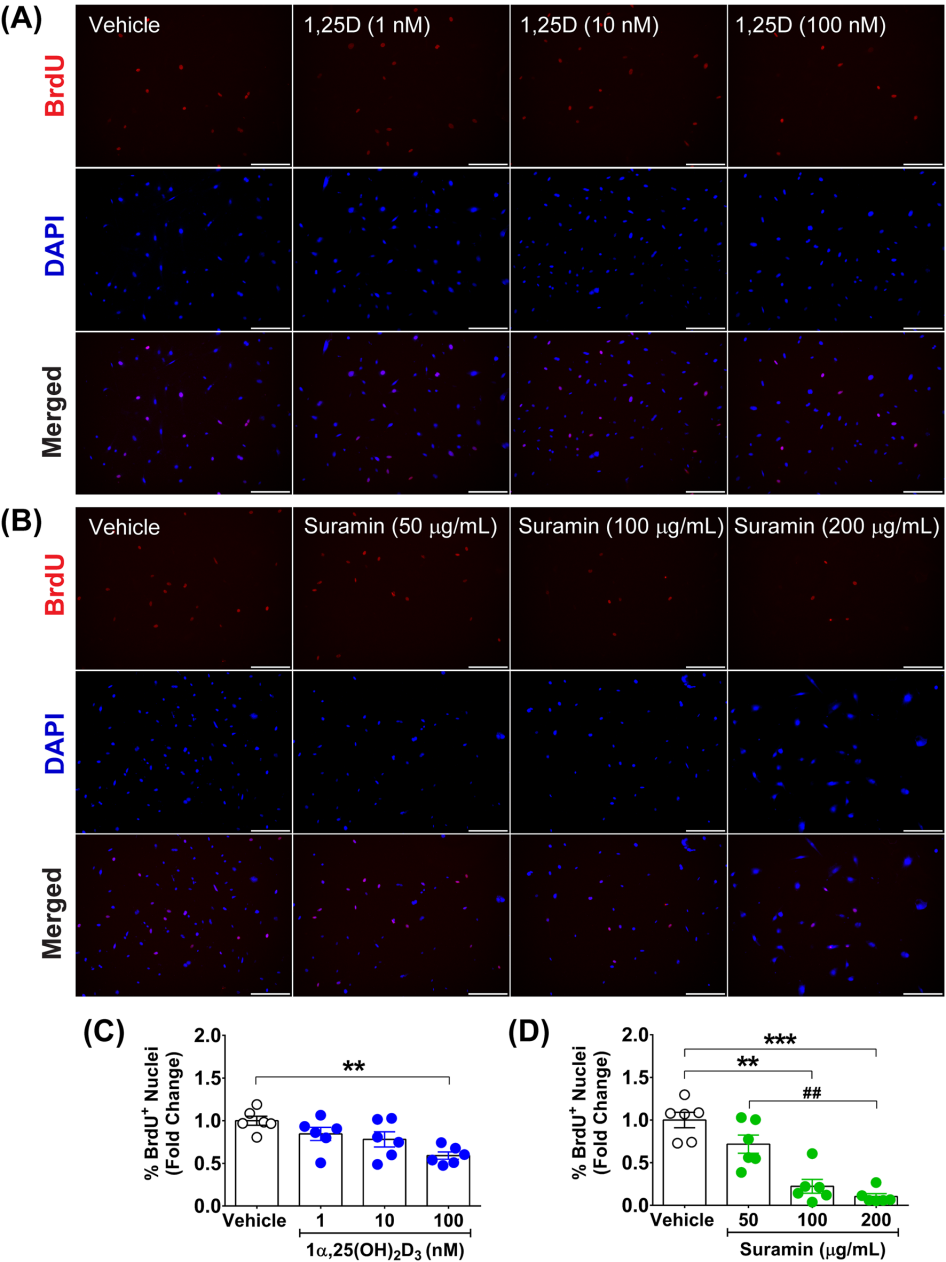
Antiproliferative effect of calcitriol and suramin on primary fibroblasts. (**A**,**B**) Representative images of BrdU staining in primary fibroblasts after daily treatment for 48 h with calcitriol at 1, 10, and 100 nM or suramin at 50, 100, and 200 µg/mL. (**C**,**D**) Quantitative analysis of BrdU^+^ nuclei (means ± SEM) (n = 6 biological replicates) after primary fibroblasts were daily treated for 48 h with calcitriol or suramin compared with vehicles. **p < 0.01, ***p < 0.001 compared with vehicles and ^##^*p* < 0.01 compared with 50 µg/mL suramin. Images were taken at magnification × 100, scale bars = 200 µm.

To evaluate the effects of calcitriol and suramin (an antifibrotic agent) on primary fibroblast proliferation that contributes to fibrogenesis, we treated cells daily with calcitriol at 1, 10, and 100 nM or suramin at 50, 100, and 200 µg/mL for 48 h. Thereafter, primary fibroblasts were pulse-labelled with BrdU to investigate the percentage of cell division at the S-phase. BrdU^+^ nuclei were significantly decreased at 48 h after calcitriol and suramin treatments, as illustrated in Fig. 2A,B. Quantitative analysis revealed that calcitriol at 100 nM significantly suppressed cell proliferation compared with the vehicle (0.59 ± 0.04- vs. 1.00 ± 0.05-fold) (*p* < 0.01) (Fig. 2C). In contrast, suramin strongly inhibited cell proliferation at concentrations of 100 µg/mL (0.22 ± 0.08-fold) (*p* < 0.01) and 200 µg/mL (0.10 ± 0.03-fold) (*p* < 0.001) compared with the vehicle (1.00 ± 0.09-fold) (Fig. 2D). These results indicate that calcitriol has the ability to suppress primary fibroblast proliferation, but its antiproliferative effect is less potent than that of the effective concentrations of suramin.

### TGF-β1 treatment abolished the antiproliferative effect of calcitriol but not suramin

**Figure 3:**
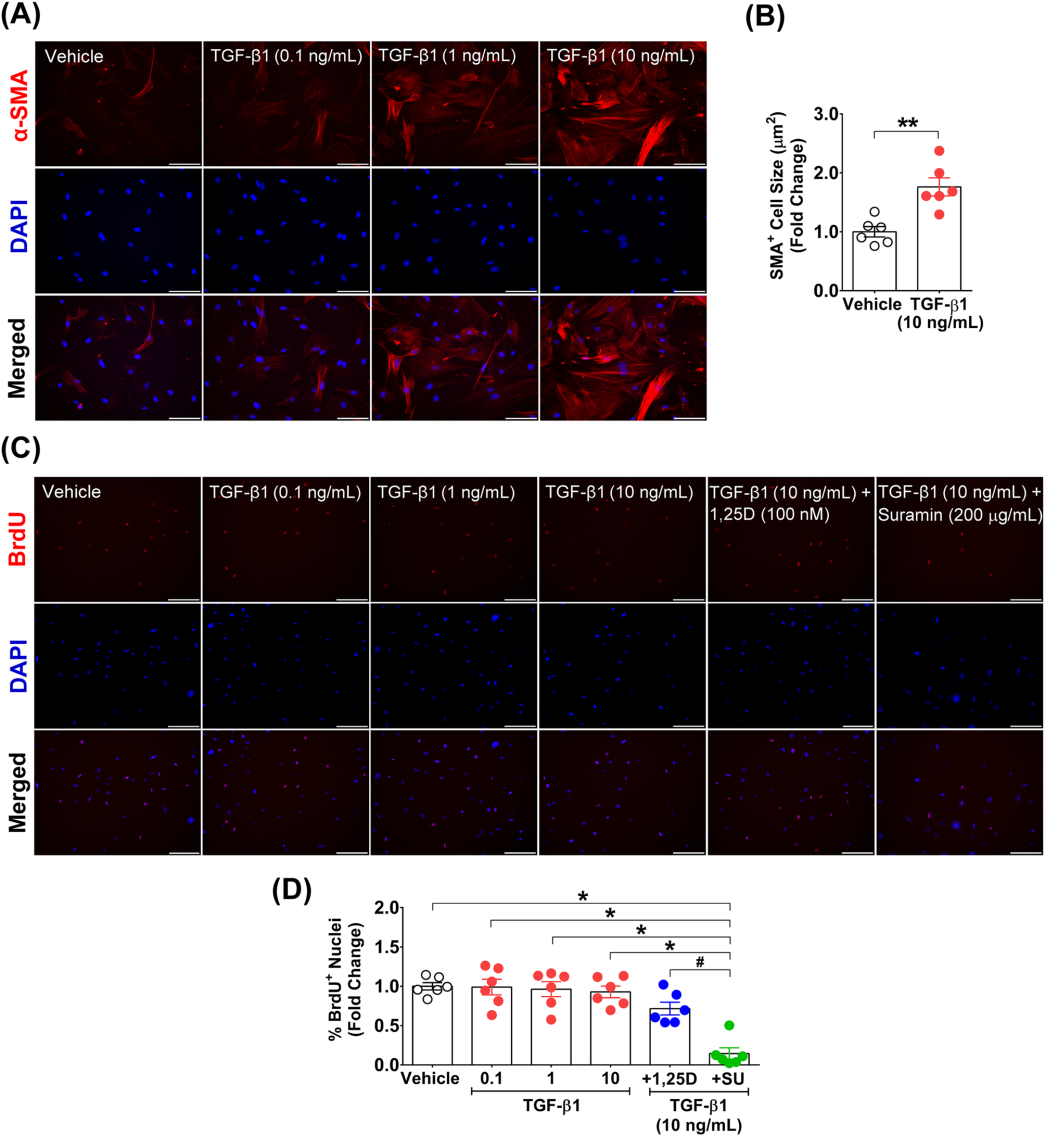
TGF-β1 stimulation abolished the antiproliferative effect of calcitriol. Representative images of (**A**) α-SMA protein expression after primary fibroblasts were treated daily for 48 h with vehicle and TGF-β1 (0.1, 1, and 10 ng/mL). (**B**) Quantitative analysis of α-SMA^+^ cell size after treatment with 10 ng/mL TGF-β1 compared with vehicle (n = 6 biological replicates). **p < 0.01 compared with the vehicle-treated group. (**C**,**D**) Representative images and quantitative analysis of BrdU^+^ nuclei after primary fibroblasts were treated daily for 48 h with TGF-β1 (0.1, 1, and 10 ng/mL), 10 ng/mL TGF-β1 plus 100 nM calcitriol, and 10 ng/mL TGF-β1 plus 200 µg/mL suramin compared with vehicle treatment (n = 6 biological replicates). Images were taken at magnifications of × 200 and × 100 (scale bars = 100 and 200 µm) for α-SMA and BrdU, respectively. *p < 0.05 and ^#^*p* < 0.05 compared with 10 ng/mL TGF-β1 plus 200 µg/mL suramin treatment.

Fibroblast to myofibroblast transition induced via TGF-β stimulation is a crucial process for ECM remodelling during skeletal muscle regeneration and fibrosis development after injury. Herein, mouse TGF-β1 recombinant protein at concentrations of 0.1, 1, and 10 ng/mL was tested to verify the effect on the induction of α-SMA protein expression that was upregulated during the transition of fibroblasts to myofibroblasts. The results revealed that mouse TGF-β1 recombinant protein at a concentration of 10 ng/mL effectively increased α-SMA protein expression in primary fibroblasts derived from skeletal muscle (Fig. 3A). This change was associated with an increase in α-SMA^+^ cell size in the TGF-β1-treated group compared with the vehicle-treated group (1.76 ± 0.15- vs. 1.00 ± 0.09-fold) (*p* < 0.01) (Fig. 3B). Nevertheless, none of the tested concentrations of TGF-β1 affected cell proliferation within 48 h after treatment according to the BrdU incorporation assay (Fig. 3C). Quantitative analysis revealed that there were no significant differences in the percentage of BrdU^+^ nuclei between the vehicle-treated group (1.00 ± 0.05-fold) and the TGF-β1-treated group at 0.1 ng/mL (0.99 ± 0.10-fold), 1 ng/mL (0.96 ± 0.10-fold), and 10 ng/mL (0.93 ± 0.07-fold) (*p* > 0.05) (Fig. 3D). Intriguingly, the antiproliferative effects of calcitriol and suramin in response to the effective dose of TGF-β1 (10 ng/mL) stimulation to induce fibroblast to myofibroblast transition were dissimilar. Under this condition, cotreatment with TGF-β1 abolished the antiproliferative effect of calcitriol (100 nM) (0.72 ± 0.08-fold) (*p* > 0.05) but not suramin (200 µg/mL) (0.14 ± 0.07-fold) (*p* < 0.05) when compared with TGF-β1 treatment alone (Fig. 3D). These results suggest that TGF-β1 stimulation modulates the antiproliferative effect of calcitriol during fibroblast to myofibroblast transition.

### Calcitriol treatment suppressed α-SMA protein expression and Smad2/3 signalling in myofibroblasts

**Figure 4:**
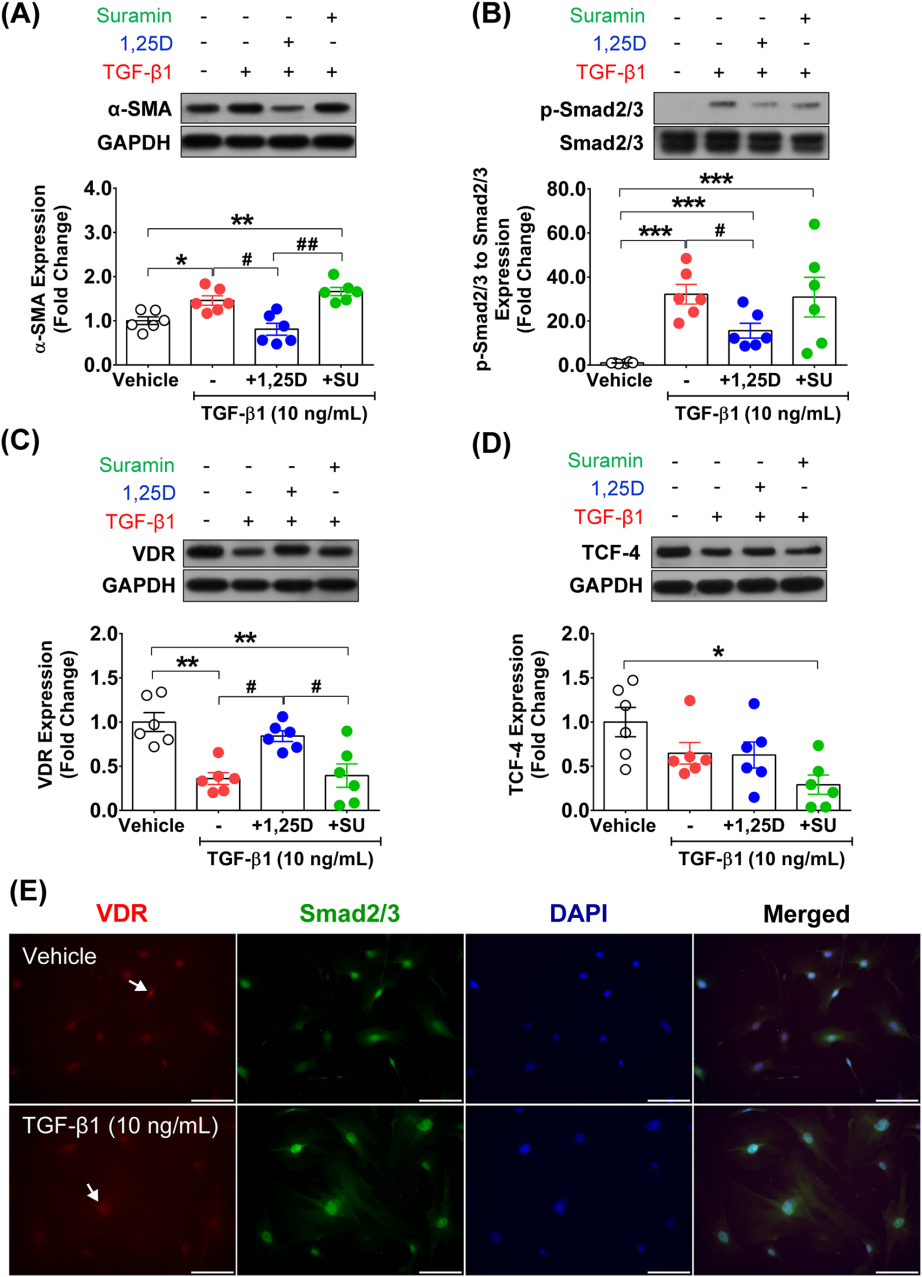
Differential effects of calcitriol and suramin on the regulation of fibrogenesis under TGF-β1 stimulation. Representative blots and quantitative analysis of (**A**) α-SMA, (**B**) phospho-Smad2/3 to Smad2/3, (**C**) VDR, and (**D**) TCF-4 protein expression after primary fibroblasts were treated daily for 48 h with vehicle, 10 ng/mL TGF-β1, 10 ng/mL TGF-β1 plus 100 nM calcitriol, and 10 ng/mL TGF-β1 plus 200 µg/mL suramin (means ± SEM) (n = 6 biological replicates). The representative blots were cropped from original blots as illustrated in the supplementary information file. The investigated protein expression levels were normalized to GAPDH or Smad2/3 protein expression obtained from the same gel and experiment. (**A**) *p < 0.05 compared with vehicle, ^#^*p* < 0.01 compared with 10 ng/mL TGF-β1, **p < 0.01 and ^##^*p* < 0.001 compared with vehicle and 10 ng/mL TGF-β1 plus 100 nM calcitriol, respectively. (**B**) ***p < 0.001 and ^#^*p* < 0.05 compared with vehicle and 10 ng/mL TGF-β1, respectively. (**C**) **p < 0.01 compared with vehicle and ^#^*p* < 0.05 compared with 10 ng/mL TGF-β1 and 10 ng/mL TGF-β1 plus 200 µg/mL suramin. (**D**) *p < 0.05 compared with vehicle. (**E**) Representative images of VDR and Smad2/3 protein expression after primary fibroblasts were treated daily with vehicle or 10 ng/mL TGF-β1 for 48 h. Images were taken at magnification × 200, scale bars = 100 µm. Arrows indicate nuclear localization of VDR protein expression.

Although the effect of calcitriol on cell proliferation was obliterated under TGF-β1 stimulation, its effect on the target proteins related to fibroblast to myofibroblast transition (α-SMA) and fibrogenesis (Smad2/3 signalling) was apparent. α-SMA protein expression was significantly increased in the TGF-β1-treated group compared with the vehicle-treated group (*p* < 0.05). Nevertheless, TGF-β1 plus calcitriol treatment reduced α-SMA protein expression compared with TGF-β1 treatment alone (0.81 ± 0.13- vs. 1.46 ± 0.11-fold) (*p* < 0.01) (Fig. 4A). To support this notion, phospho-Smad2/3 significantly increased under TGF-β1 stimulation (32.16 ± 4.47-fold) (*p* < 0.001), and its expression was significantly suppressed with TGF-β1 plus calcitriol treatment (15.64 ± 3.33-fold) (*p* < 0.05) (Fig. 4B).

Notably, the antifibrogenic effect of calcitriol was associated with a significant increase in VDR protein expression (0.84 ± 0.06-fold) (*p* < 0.05) that was significantly suppressed under TGF-β1 stimulation (0.36 ± 0.07-fold) (*p* < 0.01) compared with that of the vehicle-treated group (1.00 ± 0.11-fold) (Fig. 4C). This result suggests that VDR may be responsible for attenuating the upregulation of phospho-Smad2/3 after TGF-β1 stimulation (Fig. 4B). Additionally, the suppression of VDR protein expression in Smad2/3^+^ myofibroblasts after TGF-β1 stimulation is illustrated in Fig. 4E. These findings indicate crosstalk between TGF-β1 stimulation and the regulation of VDR protein expression in skeletal muscle primary fibroblasts. Nevertheless, calcitriol treatment had no effect on the TCF-4 transcription factor, which regulates fibrogenic determination (*p* > 0.05) (Fig. 4D). Altogether, calcitriol treatment could upregulate VDR protein expression to suppress fibroblast to myofibroblast transition and Smad2/3 signalling-associated fibrogenesis under TGF-β1 stimulation without affecting fibrogenic determination.

### Differential effects of calcitriol and suramin on the suppression of α-SMA protein expression and Smad2/3 signalling under TGF-β1 stimulation

In contrast to calcitriol, suramin treatment under TGF-β1 stimulation significantly increased α-SMA protein expression compared with vehicle treatment (1.66 ± 0.09- vs. 1.00 ± 0.09-fold) (*p* < 0.001) (Fig. 4A). No significant differences in the relative expression level of phospho-Smad2/3 to Smad2/3 protein in the TGF-β1 plus suramin-treated group compared with the TGF-β1-treated group (*p* > 0.05) were observed (Fig. 4B). Nevertheless, TGF-β1 plus suramin treatment significantly decreased TCF-4 protein expression compared with that in the vehicle-treated group (0.29 ± 0.11- vs. 1.00 ± 0.17-fold) (*p* < 0.05) (Fig. 4D).

**Figure 5:**
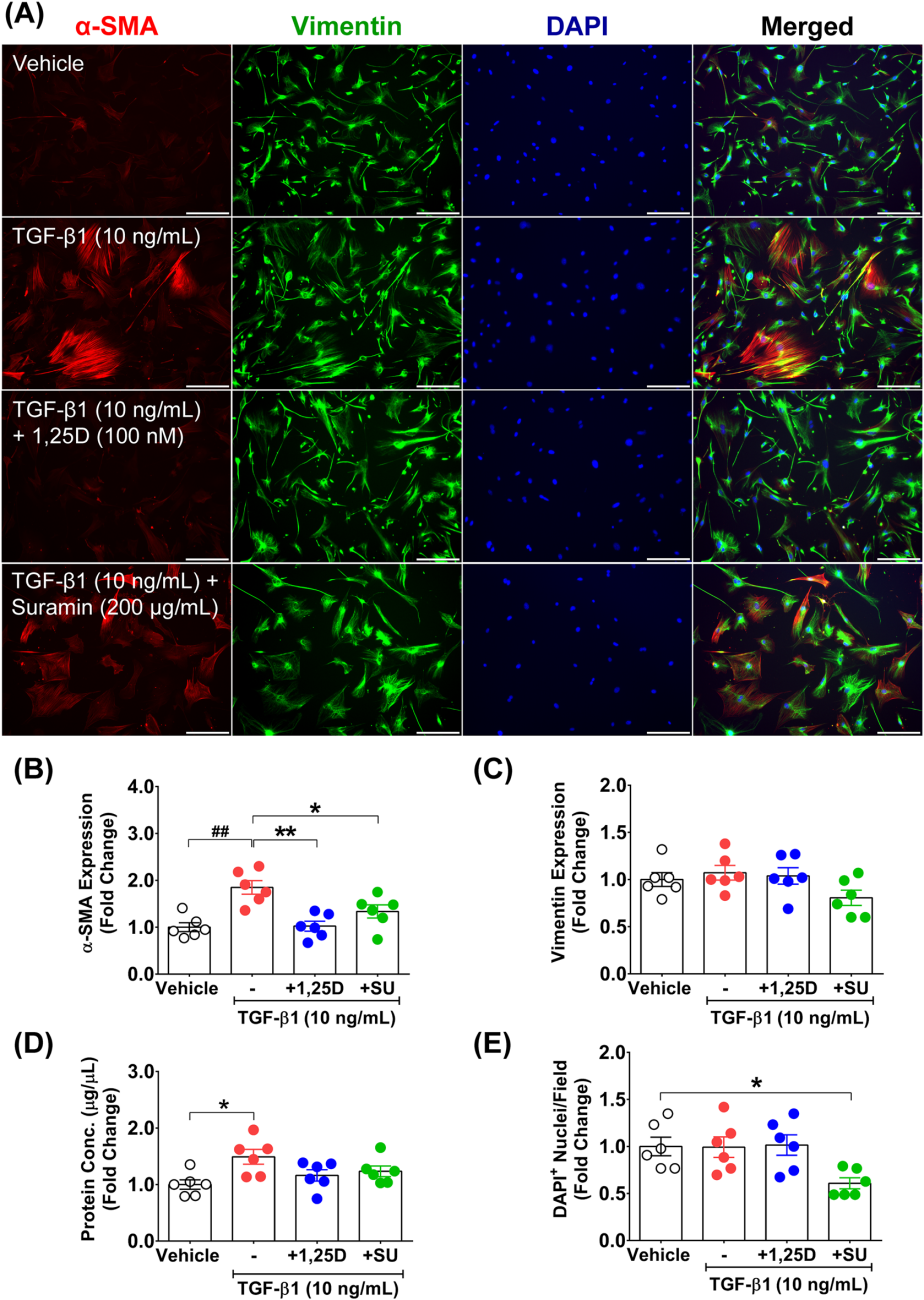
α-SMA/vimentin protein expression, protein concentration, and cell number after calcitriol and suramin treatments under TGF-β1 stimulation. (**A**) Representative images of α-SMA and intermediate filament vimentin protein expression after primary fibroblasts were treated daily for 48 h with 10 ng/mL TGF-β1, 10 ng/mL TGF-β1 plus 100 nM calcitriol, and 10 ng/mL TGF-β1 plus 200 µg/mL suramin compared with vehicle. Images were taken at magnification × 100, scale bars = 200 µm. (**B**,**C**) Quantitative analysis of α-SMA/vimentin protein expression, (**D**) protein concentration, and (**E**) DAPI^+^ nuclei/field after primary fibroblasts were treated daily for 48 h with 10 ng/mL TGF-β1, 10 ng/mL TGF-β1 plus 100 nM calcitriol, and 10 ng/mL TGF-β1 plus 200 µg/mL suramin compared with vehicle. (**B**) ^##^*p* < 0.001 compared with vehicle and *p < 0.05, **p < 0.01 compared with 10 ng/mL TGF-β1 and (**D**,**E**) *p < 0.05 compared with vehicle (means ± SEM) (n = 6 biological replicates).

To clarify how suramin exerts a differential antifibrogenic effect compared with calcitriol under TGF-β1 stimulation, we used immunocytochemical analysis to investigate α-SMA and intermediate filament vimentin protein expression levels accompanied by cell number quantification (Fig. 5A). The results demonstrated that TGF-β1 plus suramin treatment significantly suppressed α-SMA protein expression level compared with TGF-β1 treatment alone (1.34 ± 0.14- vs. 1.85 ± 0.14-fold) (*p* < 0.05) (Fig. 5B). This suppressive effect of suramin on α-SMA protein expression did not significantly affect vimentin protein expression (Fig. 5C) or protein concentration (Fig. 5D) in TGF-β1-treated cells. However, the effect of suramin but not calcitriol was associated with a significant decrease in cell number compared with that of the vehicle-treated group (0.61 ± 0.06- vs. 1.00 ± 0.10-fold) (*p* < 0.05) (Fig. 5E). These findings suggest that the potent inhibitory effect of suramin on primary fibroblast proliferation could lead to cell stress and affect α-SMA protein expression quantification when relying on a total protein analysis technique, e.g., western blotting. Thus, suramin could elicit its effect on the suppression of fibrogenesis mainly via decreased cell proliferation associated with lowering α-SMA protein expression and modulating the expression of transcription factor that regulates fibrogenic determination.

### *Vdr* gene silencing effectively suppressed VDR protein expression in primary fibroblasts

**Figure 6:**
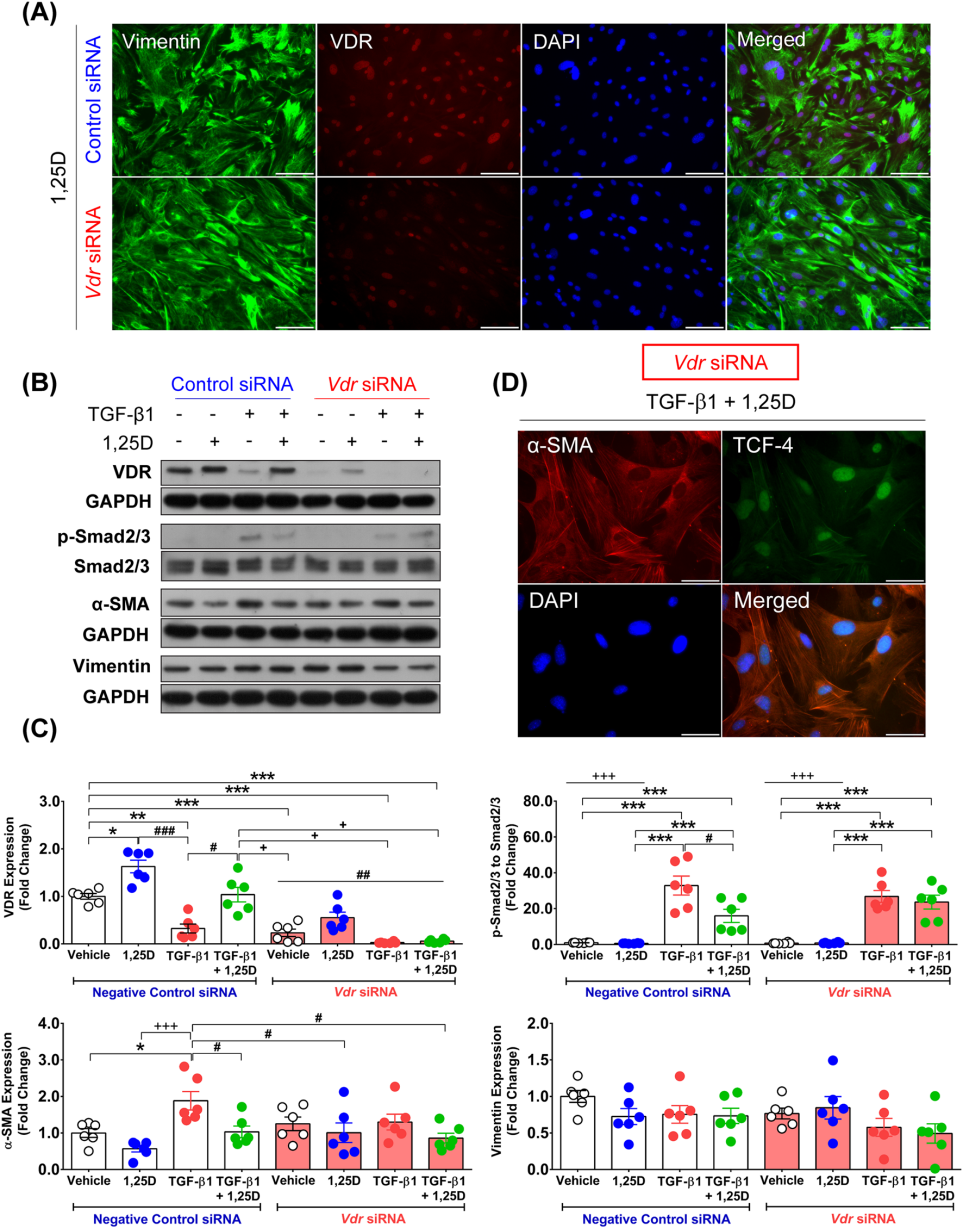
VDR-dependent activation required for calcitriol action on primary fibroblasts. (**A**) Representative images of intermediate filament vimentin and VDR protein expression in primary fibroblasts transfected with negative control/*Vdr* siRNA and daily treated for 48 h with 100 nM calcitriol. Images were taken at magnification × 200, scale bars = 100 µm. (**B**) Representative blots and (**C**) quantitative analysis of VDR, phospho-Smad2/3 to Smad2/3, α-SMA**,** and vimentin protein expression after primary fibroblasts were transfected with negative control/*Vdr* siRNA before daily treatment for 48 h with vehicle, 100 nM calcitriol, 10 ng/mL TGF-β1, and 10 ng/mL TGF-β1 plus 100 nM calcitriol (means ± SEM) (n = 6 biological replicates). The representative blots were cropped from original blots as illustrated in the supplementary information file. The investigated protein expression levels were normalized to GAPDH or Smad2/3 protein expression obtained from the same gel and experiment. VDR: *p < 0.05, **p < 0.01, ***p < 0.001 compared with vehicle, ^+^*p* < 0.05 compared with 10 ng/mL TGF-β1 plus calcitriol, ^#^*p* < 0.05, ^###^*p* < 0.001 compared with 10 ng/mL TGF-β1, and ^##^*p* < 0.01 compared with calcitriol under negative control siRNA transfection conditions. Phospho-Smad2/3 to Smad2/3: ***p < 0.001 compared with vehicle and calcitriol under negative control siRNA and *Vdr* siRNA-transfected conditions, ^#^*p* < 0.05 compared with 10 ng/mL TGF-β1 under negative control siRNA-transfected condition, and ^+++^*p* < 0.001 compared with 10 ng/mL TGF-β1 and 10 ng/mL TGF-β1 plus calcitriol across groups (negative control/*Vdr* siRNA transfected condition). α-SMA: *p < 0.05 compared with vehicle, ^+++^*p* < 0.001 compared with calcitriol, and ^#^*p* < 0.05 compared with 10 ng/mL TGF-β1 under negative control siRNA transfection conditions. (**D**) Representative images of α-SMA and TCF-4 protein expression after daily treatment for 48 h with 10 ng/mL TGF-β1 plus 100 nM calcitriol in primary fibroblasts transfected with *Vdr* siRNA. Images were taken at magnification × 400, scale bars = 50 µm.

To verify the contribution of VDR activation in response to calcitriol treatment to the suppression of fibrogenesis process, we performed RNA interference targeting of the *Vdr* gene to delineate the effects of calcitriol on primary fibroblasts. *Vdr* siRNA transfection significantly decreased VDR protein expression in primary fibroblasts under calcitriol treatment, as illustrated in Fig. 6A,B. Quantitative analysis revealed that the endogenous VDR protein expression level after *Vdr* siRNA transfection was significantly decreased in the vehicle-treated primary fibroblasts (0.23 ± 0.08-fold) (*p* < 0.001) and myofibroblasts under TGF-β1 stimulation (0.03 ± 0.01-fold) (*p* < 0.001) compared with the negative control siRNA-transfected cells (Fig. 6C). Additionally, *Vdr* gene silencing significantly suppressed VDR protein expression in the calcitriol-treated primary fibroblasts (1.63 ± 0.13- vs. 0.55 ± 0.12-fold) and calcitriol-treated myofibroblasts under TGF-β1 stimulation (1.04 ± 0.15- vs. 0.06 ± 0.01-fold) when compared between the negative control siRNA- and *Vdr* siRNA-transfected conditions (Fig. 6C). Altogether, these results suggest that VDR protein expression was effectively suppressed after *Vdr* gene silencing in this study.

### *Vdr* gene silencing diminished the effects of calcitriol on primary fibroblasts

Next, the impact of *Vdr* gene silencing on the transition of primary fibroblasts to myofibroblasts and Smad2/3 signalling-associated fibrogenesis was investigated. The suppressive effect of calcitriol on the reduction in α-SMA and phospho-Smad2/3 protein expression levels after cotreatment with TGF-β1 was diminished without affecting vimentin protein expression in the *Vdr* siRNA-transfected condition compared with the negative control siRNA-transfected condition (*p* > 0.05) (Fig. 6B,C). In support of this notion, apparent of TCF-4^+^ myofibroblasts after cotreatment with TGF-β1 plus calcitriol in *Vdr* siRNA-transfected condition was evident (Fig. 6D). Collectively, these findings affirm that the suppressive effect of calcitriol on fibroblast to myofibroblast transition and Smad2/3 signalling-associated fibrogenesis requires VDR activation.

## Discussion

This study aimed to investigate the effects of calcitriol on skeletal muscle primary fibroblasts and signalling-associated fibrogenesis. The main findings are that calcitriol has suppressive effects on primary fibroblast proliferation, the transition of fibroblasts to myofibroblasts, and Smad2/3 signalling-associated fibrogenesis. Nevertheless, the antiproliferative effect of calcitriol on primary fibroblasts was diminished under TGF-β1 stimulation, which suppressed VDR activation. Although calcitriol treatment was able to suppress α-SMA protein expression and Smad2/3 signalling in myofibroblasts, *Vdr* gene silencing obliterated its antifibrogenic effect. Moreover, calcitriol and the antifibrotic agent suramin have differential effects on the inhibition of fibrogenesis under TGF-β1 stimulation.

Calcitriol, an active form of vitamin D, has been reported to modulate gene transcription, cellular function, and signalling-associated fibrogenesis in the kidney^34,35^, lung^29^, and liver^36^. These findings suggest noncalcaemic effects of calcitriol that could contribute to the regulation of fibrogenic process. In this study, the antiproliferative effect of calcitriol on skeletal muscle primary fibroblasts was in accordance with a previous report that calcitriol was able to suppress cell proliferation by increasing VDR expression in lung fibroblasts^29^ and intestinal fibroblasts^37^. In support of this notion, the inhibitory effect of calcitriol on fibroblast proliferation could be related to decreased cell cycle regulatory genes^38^. In contrast to calcitriol, an inhibitory effect of suramin on skeletal muscle primary fibroblast proliferation under TGF-β1 stimulation was apparent. This finding supports an antifibrotic effect of suramin on skeletal muscle, which is a tissue susceptible to fibrosis development in response to TGF-β1 stimulation^39^. Suramin can act as a TGF-β receptor antagonist in fibroblasts via suppression of TGF-β1 signalling cascades^26^. In addition, an inhibitory effect of suramin on fibroblast proliferation could be related to suppression of purinergic P2Y receptor (ATP receptor) expression^40^. These underlying mechanisms might contribute to the differential effects of suramin and calcitriol on the suppression of fibroblast proliferation under TGF-β1 stimulation.

Fibroblast to myofibroblast transition in skeletal muscle is a crucial process for fibrogenesis and is regulated via TGF-β/Smad signalling^26^. Herein, the crosstalk between TGF-β1 stimulation and suppression of VDR protein expression in primary fibroblasts derived from skeletal muscle was evident. This suppressive effect of TGF-β1 could be associated with the reduction in unliganded VDR basal activity, which has been proposed to regulate a broad range of genes in fibroblasts^38^. Additionally, the suppression of VDR protein expression after TGF-β stimulation has been reported in dermal fibroblasts^41^ and lung fibroblasts^42^, which also play a crucial role in the development of tissue fibrosis. The potential mechanism of the crosstalk between TGF-β signalling and VDR regulation could be related to an inhibitory effect of TGF-β on VDR that acts as a negative regulator to block Smad-dependent transcription^41^. Therefore, the suppression of VDR protein expression in fibroblasts could be associated with an initial event of fibrogenesis in response to TGF-β1 stimulation.

Although basal VDR activity was suppressed under TGF-β1 stimulation, cotreatment of calcitriol with TGF-β1 rescued VDR expression level that subsequently decreased α-SMA protein expression and Smad2/3 signalling in myofibroblasts. This inhibitory effect of calcitriol on the fibroblast to myofibroblast transition and Smad signalling-associated fibrogenesis was in accordance with previous reports in cardiac fibroblasts^43^, lung fibroblasts^42^ and nasal polyp-derived fibroblasts^44^. The suppressive effect of calcitriol on TGF-β1/Smad signalling might be involved in the disruption of Smad3 binding to DNA via a VDR-dependent mechanism that subsequently suppresses the expression of the *Acta2* gene encoding α-SMA^34^. Nevertheless, this antifibrogenic effect of calcitriol on the inhibition of fibrogenesis required a supraphysiological dose that did not reflect its physiological level. Notably, an antifibrogenic effect of calcitriol on skeletal muscle primary fibroblasts was obliterated after *Vdr* gene silencing in this study, supporting its action through a genomic mechanism that requires cognate receptor activation. In contrast to calcitriol, suramin strongly inhibited cell proliferation associated with an induction of α-SMA and suppression of TCF-4 protein expression in myofibroblasts. These findings indicate that the antifibrogenic effect of suramin involves its potent antiproliferative effect and suppression of transcription factor related to fibrogenic determination of fibroblasts rather than its action on inhibition of α-SMA protein expression and Smad2/3 signalling-associated fibrogenesis.

Although an antifibrogenic effect of calcitriol on cultured skeletal muscle primary fibroblasts in vitro was demonstrated in this study, its antiproliferative effect could be suppressed under TGF-β1 stimulation. This modulating effect of TGF-β1, which serves as a profibrotic factor, might affect calcitriol action on the suppression of fibroblast proliferation in regenerating muscle after injury in vivo. Previously, TGF-β1 protein expression was reported to be substantially increased during the early phase of regeneration after muscle injury^45^. Additionally, daily intramuscular administration of calcitriol at this phase of regeneration that highly expressed TGF-β1 protein significantly increased vimentin protein expression and subsequently muscular fibrosis in regenerating muscle^14^. This induction of muscular fibrosis could be related to an imbalance between the interplay of fibroblast and SMSC populations in which differentiated SMSCs were significantly decreased after intramuscular administration of calcitriol into regenerating muscle^14^. This decline in the differentiation of SMSCs after calcitriol administration might be associated with an induction of the self-renewal process in the SMSC population via Notch and FOXO3 signalling^46^. Therefore, an antifibrogenic effect of calcitriol in vivo requires future research to optimize the treatment strategy to elicit the desired effect on suppression of fibrogenesis without affecting SMSC function.

Generally, calcitriol level in humans is tightly regulated at low concentration in the blood, and its concentration might not correlate well with overall vitamin D status. In contrast, the major circulating blood vitamin D [calcifediol, 25(OH)D] level has been reported to be associated with muscle strength and physical performance in healthy individuals^47^, community-dwelling older adults^48^, and athletes^49^. In addition, low blood 25(OH)D levels are associated with low VDR protein expression in skeletal muscle in both humans^50^ and rodents^16^. Moreover, 25(OH)D can be metabolized in the kidney and extrarenal tissues via 1α-hydroxylase to convert to 1α,25(OH)_2_D, which acts through VDR activation^51^. Although previous in vitro studies demonstrated that SMSCs could be the cellular source of VDR in skeletal muscle^16,46,50^, none of the studies investigated the impact of VDR and calcitriol action in skeletal muscle primary fibroblasts. In this study, *Vdr* gene silencing downregulated VDR protein expression in primary fibroblasts, resulting in an abolished antifibrogenic effect of calcitriol. This suppressive effect could imitate vitamin D deficiency [25(OH)D < 20 ng/mL]^52^ conditions associated with low VDR protein expression levels in skeletal muscle^16,50^. This finding suggests the potential contribution of low blood vitamin D and VDR expression levels to the responsiveness of fibroblasts to calcitriol for alleviation of muscular fibrosis in elderly and athletic populations that are susceptible to fibrosis development after skeletal muscle injury^53^. Therefore, maintaining blood vitamin D at the physiological level [25(OH)D ≥ 30 ng/mL]^52^ would increase the effectiveness of the antifibrogenic effect of calcitriol to counteract improper fibrogenesis that could impair the regenerative ability of skeletal muscle after injury.

## Conclusion

Calcitriol has the ability to suppress skeletal muscle primary fibroblast proliferation and target protein-associated fibrogenesis. However, its antifibrogenic effects could be modulated via TGF-β1 and VDR-dependent activation. Therefore, strategies to minimize the crosstalk of calcitriol with TGF-β1 and optimize VDR protein expression level are essential for the effectiveness of calcitriol in the regulation of fibrogenesis during skeletal muscle regeneration after injury.

## Materials and methods

### Animals

Male C57BL/6 mice were purchased from Nomura Siam International Co, Ltd. (Bangkok, Thailand). Mice were acclimatized for 1 week before conducting the study at the Central Animal Facility (CAF), Faculty of Science, Mahidol University, Thailand. During the acclimatization period, mice received the standard diet and were housed in an environmentally controlled room with a temperature of 22 ± 1 °C and a standard light cycle (12:12 h). Male mice were used in this study to compare the effects of calcitriol on cultured skeletal muscle primary fibroblasts and fibrogenesis-related processes with our previous in vivo study using a skeletal muscle regeneration model^14^.

### Primary fibroblast isolation, characterization, and culture

Mice (aged 1 month old) were anaesthetized with isoflurane, and the hindlimb muscles (gastrocnemius, soleus, plantaris, and tibialis anterior) were dissected for primary fibroblast isolation using aseptic techniques. Briefly, the dissected hindlimb muscles were minced into small pieces and incubated in Dulbecco's modified Eagle’s medium (DMEM) containing 0.1% PRONASE® Protease, *Streptomyces griseus* (53702) (Merck Millipore, MA, USA) using a 35-mm Petri dish at 37 °C and 5% CO_2_ (60 min) for the enzymatic dissociation process. Thereafter, PRONASE® Protease solution containing muscle fragments was centrifuged to collect the muscle fragments, which were resuspended in DMEM + 20% foetal bovine serum (FBS) (10270-106) (Gibco, NY, USA). After vigorous trituration and time to allow the muscle fragments to settle down, the cell suspension was transferred and filtered through a 40-µm cell strainer and then centrifuged to collect pellets containing isolated cells. When the isolation processes were finished, a preplating technique was used to allow primary fibroblasts that demonstrated adherent characteristics to attach after an initial plating for 1 h in uncoated 35-mm Petri dish. Primary fibroblasts were cultured for cell expansion in DMEM with 20% FBS, 1% penicillin‒streptomycin (15140-122) (Gibco, NY, USA), and 5 ng/mL basic fibroblast growth factor (bFGF) protein, human recombinant (GF003) (Merck Millipore, MA, USA). For confirmation of the nonmyogenic population of these mesodermal lineage-derived cells, vimentin^+^/TCF-4^+^ primary fibroblasts were cultured in DMEM with 2% horse serum (16050-130) (Gibco, NY, USA) and 1% penicillin‒streptomycin for 48 h to induce myogenic differentiation, and no expression of embryonic myosin heavy chain (EbMHC)/myosin heavy chain (MHC) proteins was detected. Cultured primary fibroblasts at passages 1–3 were used for experiments in this study.

### Assessment of calcitriol action on primary fibroblast proliferation, transition to myofibroblasts, and the TGF-β/Smad signalling cascade

Primary fibroblasts were cultured in 2% gelatine-coated 12-well plates using DMEM with 20% FBS, 1% penicillin‒streptomycin, and 5 ng/mL bFGF for 24 h, and then, the culture media were changed to 10% FBS and 1% penicillin‒streptomycin before the experiments. For cell proliferation assessment, primary fibroblasts were treated daily with vehicle (0.1% ethanol, final concentration) or calcitriol (71820) (Cayman Chemical, MI, USA) at 1, 10, and 100 nM (final concentrations) for 48 h. Cell proliferation was assessed using a BrdU incorporation assay by pulse-labelling the cells with 5-bromo-2′-deoxyuridine (BrdU) (B5002) (Sigma-Aldrich, MO, USA). The effect of calcitriol on cell proliferation was compared with that of suramin, sodium salt (574625) (Merck Millipore, MA, USA) at concentrations of 50, 100, and 200 µg/mL (final concentrations). Sterile type I water was used to dissolve suramin and served as a vehicle for suramin treatment. Suramin was selected for use as an antifibrotic agent in this study according to a previous investigation that demonstrated the effects of suramin on the inhibition of fibroblast proliferation in vitro and alleviation of muscular fibrosis in mice^32^. The starting concentration of suramin at 50 µg/mL was based on a previous report of its inhibitory effect on NIH 3T3 fibroblast proliferation^32^.

For analysis of the fibroblast to myofibroblast transition and TGF-β/Smad signalling cascade in response to calcitriol treatment, cells were initially treated with mouse TGF-β1 recombinant protein (5231LF) (Cell Signaling Technology, MA, USA) at 0.1, 1, and 10 ng/mL (final concentrations) daily for 48 h to assess the effect of TGF-β1 on primary fibroblasts. 20 mM citrate, pH 3.0 (9871) (Cell Signaling Technology, MA, USA) was used as a vehicle for TGF-β1 treatment. For the cotreatment experiment, the effective concentration of TGF-β1 (10 ng/mL) was used for cotreatment with calcitriol or suramin daily for 48 h to investigate the modulating effect of calcitriol compared with suramin on fibroblast to myofibroblast transition and the TGF-β/Smad signalling cascade.

### BrdU incorporation assay

Primary fibroblasts were pulse-labelled with BrdU (10 µM final concentration) for 1 h at 47 h after initial treatment. For assessment of cell proliferation, cells were fixed with 4% paraformaldehyde (PFA) (15713-S) (Electron Microscopy Sciences, PA, USA) for 10 min. DNA was denatured using 2 N HCl containing 0.5% Triton X-100 (9410) (Merck Millipore, MA, USA) for 30 min at 37 °C followed by neutralization with 0.1 M borate buffer pH 8.5 for 10 min. Nonspecific staining was blocked with 5% normal goat serum (PCN5000) (Invitrogen, CA, USA) for 30 min before applying mouse monoclonal anti-BrdU antibody (1:1000, B2531) (Sigma-Aldrich, MO, USA) for 1 h. Thereafter, goat anti-mouse Alexa Fluor® 568 IgG (H + L) (1:500, A-11004) secondary antibody (Invitrogen, CA, USA) was incubated for 1 h, postfixed the cells with 4% PFA for 5 min, and stained with 4',6-diamidino-2-phenylindole, dihydrochloride (DAPI) (D1306) (Invitrogen, CA, USA) for nuclear visualization. Representative images were acquired using an Olympus Inverted Fluorescence Microscope Model IX83 (Olympus, Tokyo, Japan) equipped with ORCA-Flash 2.8 Digital CMOS Camera (C11440) (Hamamatsu Photonics, Hamamatsu, Japan). Ten images were randomized at magnification × 100 for quantitative analysis of % BrdU^+^ nuclei/total nuclei using image acquisition software (cellSens Dimension Desktop, Olympus, Japan).

### siRNA transfection

Primary fibroblasts were plated in 2% gelatine-coated 12-well plates and cultured using DMEM with 20% FBS, 1% penicillin‒streptomycin, and 5 ng/mL bFGF. After reaching 60% confluency, cells were transfected with negative control siRNA (4390843) or *Vdr* siRNA (4390771) (Assay ID: S75929) (Invitrogen, CA, USA) at a final concentration of 10 pmol using Lipofectamine RNAiMax (13778030) transfection reagent (Invitrogen, CA, USA). At the time of transfection, cultured media were removed and washed twice with Opti-MEM™ reduced serum medium (31985070) (Gibco, NY, USA) with no antibiotics. Then, Opti-MEM™ reduced serum medium with 10% FBS (no antibiotics) was added for 30 min before transfection. Lipofectamine/siRNA complexes were prepared according to the manufacturer’s instructions before being added to the cells. Cells were incubated with Lipofectamine/siRNA complexes for 6 h before culture media were replaced with fresh Opti-MEM™ reduced serum medium with 10% FBS (no antibiotics) for 18 h. Thereafter, culture media were changed to DMEM with 10% FBS and 1% penicillin‒streptomycin for conducting experiments.

### Immunocytochemistry

Cells were fixed with 4% PFA for 10 min, permeabilized with 0.1% Triton X-100 for 5 min, blocked nonspecific staining with 5% normal goat serum for 30 min, and incubated with primary antibodies as follows for 2 h: mouse monoclonal anti-VDR (D-6) (1:100, sc-13133), mouse monoclonal anti-TCF-4 (D-4) (1:100, sc-166699), mouse monoclonal anti-MyoD (G-1) (1:500, sc-377460), and mouse monoclonal anti-MYH3 (F1.652) (1:200, sc-53091) (Santa Cruz Biotechnology, CA, USA); rabbit monoclonal anti-vimentin (D21H3) XP® (1:400, 5741) and rabbit monoclonal anti-Smad2/3 (D7G7) (1:400, 8685) (Cell Signaling Technology, MA, USA); mouse monoclonal anti-myosin heavy chain antibody clone A4.1025 (1:500, 05–716) (Upstate, CA, USA); and mouse monoclonal anti-α-SMA (1:500, A2547) (Sigma-Aldrich, MO, USA). Thereafter, goat anti-rabbit Alexa Fluor® 488 IgG (H + L) (1:500, A-11008), goat anti-mouse Alexa Fluor® 568 IgG (1:500, A-11004) (H + L), goat anti-mouse Alexa Fluor® 488 IgG_1_ (1:500, A-21121), or goat anti-mouse Alexa Fluor® 568 IgG_2a_ (1:500, A-21134) (Invitrogen, CA, USA) secondary antibodies were incubated for 1 h followed by DAPI staining for nuclear visualization. Representative images were captured at magnifications of × 100, × 200, and × 400 using an Olympus Inverted Fluorescence Microscope Model IX83 (Olympus, Tokyo, Japan) equipped with ORCA-Flash 2.8 Digital CMOS Camera (C11440) (Hamamatsu Photonics, Hamamatsu, Japan). For quantitative analysis, ten images of α-SMA and vimentin staining were randomized at magnification × 100. The α-SMA^+^ cell size, expression level of α-SMA and vimentin proteins, and number of DAPI^+^ nuclei were analysed using cellSens Dimension Desktop software (Olympus).

### Western blot analysis

Cells were washed twice with ice-cold PBS, and protein samples were extracted using lysis buffer containing ice-cold RIPA buffer with protease inhibitor (P8340) (1:100) (Sigma-Aldrich, MO, USA) and phosphatase inhibitor cocktails (524625) (1:100) (Merck Millipore, MA, USA). A bicinchoninic acid (BCA) assay was used to determine protein concentrations, and serial dilutions of bovine serum albumin (12659) (Merck Millipore, MA, USA) served as standard protein concentrations to calculate the standard curve. The optical density was measured at 570 nm using an Infinite® M200 Pro microplate reader (Tecan Trading AG, Männedorf, Switzerland). Protein samples containing sample buffer were denatured by heating at 60 °C for 10 min before loading into SDS–polyacrylamide gels (4% stacking and 10% separating gels). Thereafter, protein samples were transferred to PVDF membranes before blocking with 5% nonfat milk for 1 h. Primary antibodies were applied overnight at 4 °C as follows: mouse monoclonal anti-VDR (D-6) (1:200, sc-13133) and mouse monoclonal anti-TCF-4 (D-4) (1:200, sc-166699) (Santa Cruz Biotechnology, CA, USA); rabbit monoclonal anti-vimentin (D21H3) XP® (1:1000, 5741), rabbit monoclonal anti-Smad2/3 (D7G7) (1:1000, 8685), and rabbit monoclonal anti-phospho Smad2 (Ser465/467)/Smad3 (Ser423/425) (D27F4) (1:500, 8828) (Cell Signaling Technology, MA, USA); mouse monoclonal anti-α-SMA (1:500, A2547) (Sigma-Aldrich, MO, USA); and rabbit polyclonal anti-glyceraldehyde-3-phosphate dehydrogenase (GAPDH) (1:5000, ABS16) (Merck Millipore, MA, USA). After washing with Tris-buffered saline + 0.1% Tween® 20, the membrane was incubated with secondary antibody for 1 h as follows: goat anti-rabbit peroxidase-conjugated antibody (1:7,000, AP132P) (Merck Millipore, MA, USA) or goat anti-mouse IgG peroxidase conjugate (1:10,000, 31430) (Thermo Scientific, Waltham, MA, USA). Protein bands were detected with Clarity™ Western ECL substrate (170-5060) (Bio-Rad, CA, USA) and visualized by exposing the membrane to CL-XPosure™ Film (34090) (Thermo Scientific, Waltham, MA, USA) in a dark-controlled environment. Stripping solution was applied for reprobing another set of primary and secondary antibodies if multiple target proteins had close/identical molecular weights in the blotting membrane. Protein expression levels were analysed using ImageJ software (National Institutes of Health, Bethesda, MD).

### Statistical analysis

Data are expressed as the means and standard errors of the mean (means ± SEMs). Normal distribution and homogeneity of variance were tested using Shapiro Wilk and Levene’s test, respectively. Log10 transformation was performed to normalize the distribution of the data where appropriate. Significant differences among groups were determined using independent t test, one-way ANOVA with Tukey’s (equal variances) or Games-Howell (unequal variances) post hoc tests. Statistical tests were performed using SPSS, and *p* < 0.05 represents a significant difference between groups.

### Ethical approval and informed consent

Experimental procedures in animals were performed in accordance with institutional guidelines for the care and use of laboratory animals. The animal protocol was approved by the Faculty of Science, Mahidol University-Institutional Animal Care and Use Committee (MUSC–IACUC: Protocol No. MUSC63-034-542). This study is reported in accordance with ARRIVE guidelines.

### Supplementary Information

Supplementary Figures.

## Data Availability

The datasets generated and/or analysed during the current study are available from the corresponding author on reasonable request.
