# Supplementary material for: TGF-β1 stimulation and VDR-dependent activation modulate calcitriol action on skeletal muscle fibroblasts and Smad signalling-associated fibrogenesis
Source: Sci Rep. 2023 Aug 23;13:13811. doi: 10.1038/s41598-023-40978-w (PMC10447566; doi:10.1038/s41598-023-40978-w)
Supplement: Supplementary file 1 — Supplementary Figures. [file 41598_2023_40978_MOESM1_ESM.pdf]

Supplementary Information

TGF-β1 stimulation and VDR-dependent activation modulate calcitriol action on skeletal muscle fibroblasts and Smad signalling-associated fibrogenesis

Ratchakrit Srikuea[1]*, Muthita Hirunsai[2]

[1]Department of Physiology, Faculty of Science, Mahidol University, Bangkok 10400, Thailand

[2]Department of Biopharmacy, Faculty of Pharmacy, Srinakharinwirot University, Nakhon Nayok 26120, Thailand

Address for correspondence:

Assoc. Prof. Dr. Ratchakrit Srikuea

Department of Physiology, Faculty of Science

Mahidol University, Bangkok 10400, Thailand

Tel: +66 22015518

Fax: +66 23547154

E-mail: ratchakrit.sri@mahidol.ac.th

Supplementary Figure S1. Original data representing α-SMA, p-Smad2/3, Smad2/3, VDR, TCF-4, and GAPDH protein expression in Figure 4A-D.

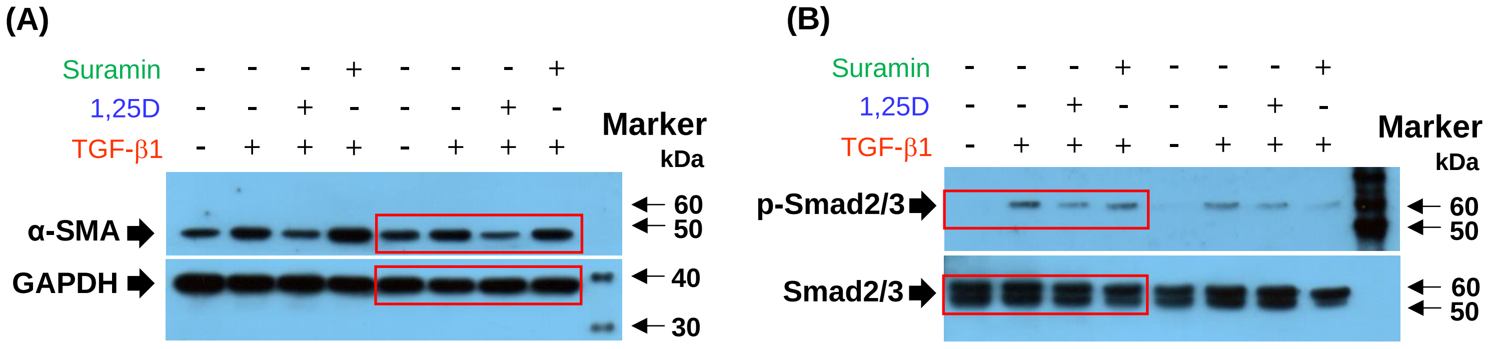

Note: (A) Membrane obtained from the same gel was cut for separately probed with the targeted proteins (α-SMA and GAPDH). (B) Membrane was initially probed with phospho-Smad2/3 antibody before stripped and reprobed with Smad2/3 antibody. Two independent biological samples were loaded and shown in the representative blots.

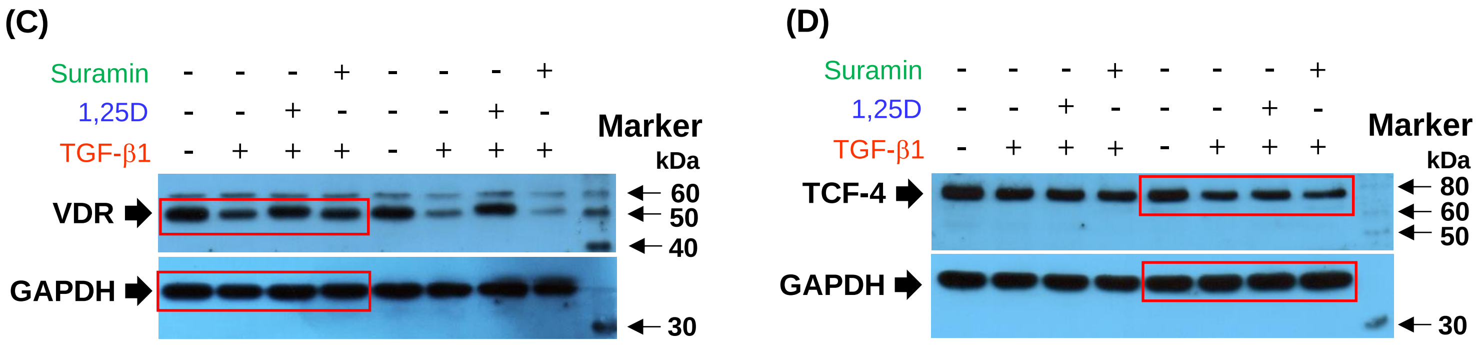

Note: Membrane obtained from the same gel was cut for separately probed with the targeted proteins (C) VDR and GAPDH and (D) TCF-4 and GAPDH. Two independent biological samples were loaded and shown in the representative blots.

Supplementary Figure S2. Original data representing VDR, p-Smad2/3, Smad2/3, α-SMA, vimentin, and GAPDH protein expression in Figure 6B.

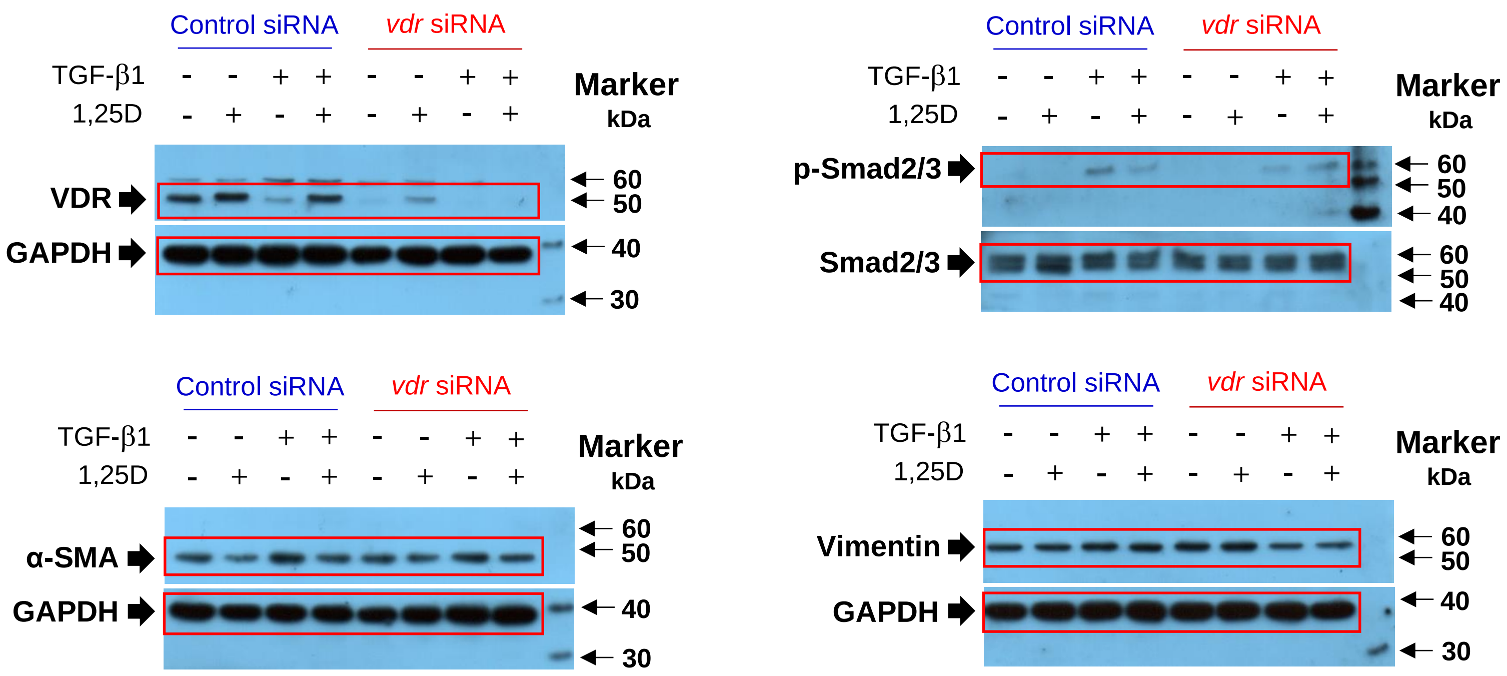

Note: Membrane obtained from the same gel was cut for separately probed with the targeted proteins (VDR, α-SMA, and vimentin) and GAPDH served as loading controls. Phospho-Smad2/3 antibody was initially probed on the membrane and membrane was stripped before reprobed with Smad2/3 antibody.
